# Toward safe dental service: risk perception and practice modification among Egyptian dental students during the COVID-19 pandemic

**DOI:** 10.1186/s12913-023-10196-1

**Published:** 2023-11-13

**Authors:** Eman Ali Younis, Abdel Aziz farouk El deep, Safynaz El Saied Shalaby, Sanaa Abd El-Fatah Abdo

**Affiliations:** Public Health and Community Medicine Department, Faculty of Medicine, Tanta University, Tanta, 31257 Egypt

**Keywords:** COVID-19, Practice, Risk perception, Dental students, Dentistry

## Abstract

**Background:**

COVID-19 was declared a major public health crisis and a challenge to healthcare systems especially dental services where dentists working close to patients face a greater risk of infection. This cross-sectional study aimed to assess the risk perception and practice modifications of undergraduate dental students at Tanta University to ensure safe dental practice during the COVID-19 pandemic.

**Methods:**

A convenience sample of 450 dental students from practical academic years at Tanta University, Egypt responded to a self-administered pre-designed validated and pretested questionnaire from the 11^th^ of February 2022 to the end of April 2022 to assess some sociodemographic data, risk perception, and their practice during the COVID-19 pandemic.

**Results:**

Females reported significantly higher levels of fear than males concerning; contracting COVID-19 infection from patients (97.3% vs. 93%) (*P* = 0.028, 95% CI = 95.6(93.2–97.3), anxiety while treating symptomatic patients (95.1% vs. 90.3%) (*P* = 0.050, 95% CI = 93.1(90.4–95.3), and fear of infecting their families (94.7% vs. 89.8%) (*P* = 0.049, 95% CI = 92.7(89.9–94.9). More than half of the students (53.7%) had good practice scores, followed by 44% with average practice and 2.2% with poor practice. A good practice score was significantly associated with the age and academic year (*P* = 0.044, *P* = 0.044). Significant predictors of a good practice score in the logistic regression analysis were; updating knowledge with current guidelines for cross-infection regarding COVID-19 (*P* = 0.001, 95% CI = 53.20, 2.733), asking every patient’s travel history before performing treatment (*P* = 0.021, 95% CI = 21.149, 1.286), deferring patients showing suspicious symptoms (*P* = 0.042, 95% CI = 20.688, 1.054), following routine universal precautions of infection for every patient (*P* = 0.016, 95% CI = 36.469, 1.438), using high-volume suction for every patient (*P* = 0.025, 95% CI = 20.826, 1.226) and using safety glasses or visor (*P* = 0.036, 95% CI = 21.673, 1.106).

**Conclusion:**

The dental students exhibited anxiety and fear while caring for patients during the COVID-19 pandemic. Additionally, considerable changes in dental practices based on the standard guidelines were observed among the students. It is strongly advised that all dental schools have student counselors who are accessible for in-person and online counseling sessions. Peer support is a great approach to spot problems with stress and anxiety in pupils and start solving them.

## Introduction

COVID-19 was detected in the city of Wuhan in China and rapidly reached all parts of the world across six continents, and this generated a major public health crisis and a challenge to healthcare systems [1, 2].

The COVID-19 pandemic has caused substantial disruption in health service delivery not only due to its direct effects but also because it pressed health systems beyond their resources indirectly exposing the existing gaps in health systems. The COVID-19 pandemic disturbed both preventive and curative services for communicable and noncommunicable diseases. Essential services have been delayed by the healthcare facilities. In addition, the COVID-19 pandemic posed a significant risk of indirect morbidity and mortality as a result of essential health services disruption [3].

The COVID-19 outbreak has shown several flaws in the dental care system, particularly concerning the inadequate coordination of pandemic-related health services and the lack of effective personal protective equipment (PPE) [4].

Healthcare workers (HCWs) and those who are in direct touch with infected persons during pandemics and natural catastrophes frequently have psychological effects. According to a study done among students, stress and worry were strongly linked to scholastic setbacks and a poor quality of life during the coronavirus epidemic [5]. Additionally, a large number of medical professionals in Wuhan reported mental health issues and anxiety disorders and said that receiving treatment for their mental health helped to relieve their symptoms [6].

A Saudi Arabian study conducted in 2021 found that throughout the epidemic, doctors, chemists, nurses, and other HCWs had a noticeably high prevalence rate of psychological illnesses. Stress, anxiety, and depression were all present in 54.6%, 60.8%, and 41.9% respectively [7].

The psychological impact on dental professionals was also investigated in Egypt where 92.6% of studied professionals were anxious about becoming infected with COVID-19 and 90.7% feared treating patients with suspicious symptoms [8]. In Egypt, from January 2020 to September 2023, there have been 516,023 confirmed cases of COVID-19 with 24,830 deaths, reported to the World Health Organization (WHO) [9].

The COVID-19 virus can be detected in the saliva of dental patients, and dentists who work close to them are at higher risk of being infected and transmitting the disease owing to the use of aerosols. Under these work circumstances, it is logical for the dentist to develop a fear of being infected by the patient [10, 11].

Several governmental dental organizations and private clinics worldwide significantly restricted the treatment of patients with dental complaints to non-deferrable urgent care after the first wave of the pandemic [12]. In China, Guo et al. (2020) reported that 94.6% of dental procedures were mainly emergency and urgent cases during the first wave of the pandemic [13].

Subsequently, based on international recommendations, the Occupational Safety and Health Administration (OSHA) suggested retaining all dental treatments not categorized as emergency or urgent dental services and postponing elective dental procedures to later [14]. These suggestions were also inspired by the recommendations of the American Dental Association, which refer to uncontrolled bleeding, soft tissue bacterial infection, intraoral or extra oral swelling, and trauma involving facial bones as dental emergencies. Severe pain caused by pulpal inflammation, dental trauma, and tooth fracture requires urgent dental care [15]. Guidelines were quickly published by the WHO and several other central institutions for treatments that could still be completed [16, 17].

Adherence to the published WHO guidelines was a major challenge for students and universities. Dental students generally face a greater risk of exposure to microorganisms and infectious diseases during their clinical terms because of their limited clinical experience and manual skills compared to trained dentists [18].

Dentists were advised to strictly adhere to standard precautions while treating patients, even those with minor dental complaints, by wearing personal protective equipment, facemasks (preferably N-95), and face shields. Additionally, adequate ventilation of the operating room, minimization of aerosol generation, and taking precautions while handling biowaste were obligatory [19]. The use of “teledentistry” or web/ telephonic consultation has been encouraged during pandemics to decrease the contact between the dentist and the patient [20].

Although the number of cases has decreased during the study execution, however, the number of cases has begun to increase these days. Therefore, concerns that threaten the existing practice must always be in the forefront of dental students' minds. It is crucial to evaluate how they perceive the risk to find any holes in current infection control procedures. Additionally, the a dearth of knowledge in this area in Egypt. So, the objectives of this study were to assess the risk perception and detect practice modifications of undergraduate dental students at Tanta University to ensure safe dental practice and reduce the risk of spreading the infection to the community.

## Methods

### Study design and settings

This is a cross-sectional study that was conducted in the Faculty of Dental Medicine at Tanta University. Tanta University is considered a great scientific edifice. It is one of the most prestigious Egyptian universities. In addition, it has a high stature among regional and world universities. It is located on El-Gaish Street. Medical Campus, Tanta in Gharbia governorate. Tanta University includes 14 faculties and institutes. The faculty of dentistry contains 11 different scientific departments. It receives great numbers of patients daily to be cured for free at the different departments of the faculty. The Bachelor's degree in Oral and Dental Medicine and Surgery is offered after a five-year full-time program of study as well as summer modules, and clinical summer training modules aimed primarily at educating and training graduates for an efficient dental practice in the new century.

### Sample size and technique

A convenient sample was chosen from 3rd, 4th, and 5th year students who practiced dental clinical work on patients.

The sample size was calculated using the Centers for Disease Control (CDC) and Prevention, Atlanta, Georgia, USA. EpiInfo 7.2.3.0 software statistical package and assuming that 50% as a proportion (p) of students modified their practice, at 95% confidence level and margin of error (d) = 5%. Based on the previous criteria, the sample size calculation was to be *n* > 384, and 10% was added to compensate for the missing data and improve validity to 424; however, 450 students completed the questionnaire who were approached by convenience sampling.

$$\mathrm{So},\mathrm{ estimated sample }(\mathrm{n}) = {(Z\alpha /2)}^{2} \times \mathrm{ P }{(1-\mathrm{ P}) \div \mathrm{ d}}^{2}= 384$$

Where Z_α/2_ is the critical value of the Normal distribution at α/2 (e.g. for a confidence level of 95%, α is 0.05 and the critical value is 1.96), d is the margin of error, and p is the sample proportion. The author disseminated 500 questionnaires. Twenty-seven were excluded for incomplete data, 450 truly completed the questionnaire and the remainder did not return the questionnaire. Total response rate was 95%. However, using a convenience sample was a limitation and a source of selection bias but we were obliged to due to the hybrid training system we tried to approach participants as much as we could to complete the required sample size.

Inclusion criteria: 3rd, 4th, and 5th year students both males and females.

Exclusion criteria: Students who do not practice clinical work on patients.

### Study tool

An English questionnaire was constructed by the authors after reviewing similar published papers [8, 21–23]. Which was developed based on the CDC Guidance for Dental Settings [24], the Clinical Management of COVID-19 Interim Guidance of the WHO [16], and the Manual of Good Practices and Biosafety of the Brazilian Federal Board of Dentistry [25]. The following data were included in the questionnaire:

1. Personal characteristics: age, sex, residence, academic year
2. Risk perception among students (11 questions closed-ended questions (yes/no)) e.g. belonging to a risk group, being afraid of getting infected, anxiety when getting in contact with treatment to a patient who is coughing, nervousness when talking to patients in close vicinity, fear of carrying the infection to their families, afraid of getting quarantined, anxiety about the cost of treatment, afraid when hearing that people are dying because of COVID-19.
3. Reasons why the dentist decided to continue the clinical work during the COVID-19 pandemic. (4 questions closed-ended questions (yes/no)
4. Practical modifications to combat the COVID-19 pandemic (15 closed-ended questions (yes/no) e.g. wearing an N-95 mask, using a rubber dam, high-volume suction, washing hands with soap and water, using FFP2/FFP3 facial filter, disposable gowns, safety glasses or visor, a rotating instrument with anti-retraction valve.

### Validity and reliability of the study tool

The validity of the questionnaire was tested by the authors via distributing hard and soft copies of the validation form together with the questionnaire to two Egyptian professors from the public health department at Tanta University Faculty of Medicine and one from the Faculty of Dentistry. The experts recommended simplifying some questions and deleting others. Regarding the time required to finish the questionnaire by participants, experts stated that all questions were understandable and participants could fill it out in 10 to 15 min.

The authors calculated the content validity index (CVI) and content validity ratio (CVR) as measurements of the content validity of the questionnaire. The individual-CVI ranged from 0.81 to 1.00, with thirty items having an I-CVI of 1.00 and four items having an I-CVI of 0.82. All items were considered relevant. The CVR was generated for each item. Twenty-nine items had a CVR of 1.00, and five had a score of 0.99.

The authors tested reliability in a pilot study by recruiting 20 students not included in the present study. We used data to assess internal consistency using Cronbach’s alpha, which was 0.793 and represented adequate internal consistency.

### Data collection

This study was conducted after its approval by the ethical committee of the faculty of medicine at Tanta University. All participating students gave valid written informed consent after a clear explanation of the study's aims and techniques.

Data were collected via a self-administered questionnaire. The questionnaire was disseminated by the researchers and well-trained fourth-year medical students at the end of clinical sessions among dentistry students at Tanta University on well-attended faculty days from the 11^th^ of February 2022 to the end of April 2022. The Faculty of Dental Medicine in Tanta was reopened at the beginning of October 2020 after a fully online academic semester program, which involved developing clinical training activities using a hybrid system (face-to-face teaching and online sessions).

### Statistical analysis

The collected data was analyzed using the Statistical Package for the Social Sciences Program (SPSS), version 25. Qualitative data was expressed as a number and percent and tested by the chi-squared test. Quantitative data was expressed as mean and standard deviation and tested by the one-way ANOVA. The crude and adjusted odds ratios using logistic regression analysis were calculated at 95% confidence intervals. Logistic regression was used to determine predictors associated with good practice. The *P* value was set to be significant at ≤ 0.05.

Scoring of practice: Practice included 15 questions answered (yes = 2 or no = 1). The total score ranged from 15 to 30 and was classified as poor practice (< 50%), average (50% -70%), and good (> 70%) based on the method described by Hashemzaei M [26].

### Ethical consideration

Ethical approval for the study was obtained from the Internal Review Board of the Faculty of Medicine, Tanta University, with code number 359241022. Consent for participation was obtained from the students after informing them about the purpose of the study. Confidentiality and privacy were maintained during the study.

## Results

The age of the study participants (*n* = 450) ranged from 20 to 24 years (mean age, 21.7 years). More than half were females and from the urban community (58.7% and 57.1% respectively). Less than half (47.6%) were in the fourth academic year, and 25.6% belonged to the risk group for coronavirus infection.

Concerning risk perception, 95.6% expressed fear of COVID-19 infection from their patients and co-workers. This percentage was significantly higher in females as compared to males (97.3%, and 93.0%, respectively) and (*P* = 0.028, 95% CI = 95.6(93.2–97.3). Most participants (93.1%) exhibited some anxiety while treating patients who presented with a cough and again females showed significantly higher perception than males (95.1%, and 90.3%, respectively) and (*P* = 0.050, 95% CI = 93.1(90.4–95.3). Most participants (92.7%) feared that they could carry the infection back to their families. Females reported significantly higher levels of that fear as compared to males (94.7%, and 89.8%, respectively) and (*P* = 0.049, 95% CI = 92.7(89.9–94.9). Regarding other items of risk perception, there was no statistically significant difference between males and females. However, 83.3% expressed discomfort and fear after hearing about the deaths of people due to COVID-19, 82.4% considered routinely using the N-90 mask in dental practice, especially during the pandemic, approximately three-quarters (74.7%) feared being quarantined if infected and 73.8% felt nervous while talking to the patients without maintaining social distancing. About two-thirds (66%) wanted to continue with the clinical procedures once the number of COVID-19 cases had declined, whereas 58.4% were worried about the cost of treatment (if infected) (Table 1).

**Table 1:** Risk perception among the studied students according to their gender (*n* = 450)

**Questions**	**Gender (Frequency of Yes answers)**						**χ**^**2**^	***P* value**	**95% CI of total** **yes percentage**
	**Female** ***n* = 264**		**Male** ***n* = 186**		**Total** ***n* = 450**				
	**n**	**%**	**n**	**%**	**n**	**%**			
**Are you afraid of getting infected with COVID-19 from a patient or co-worker**?	257	97.3	173	93.0	430	95.6	4.835	0.028*	95.6(93.2–97.3)
**Are you anxious when getting in contact with treatment for a patient who is coughing or suspected of COVID-19 during clinical training?**	251	95.1	168	90.3	419	93.1	3.843	0.050*	93.1(90.4–95.3)
**Do you want to stop your clinical course until the number of COVID-19 cases starts declining?**	180	68.2	117	62.9	297	66.0	1.355	0.244	66.0(61.4–70.4)
**Do you feel nervous when talking to patients in close vicinity?**	199	75.4	133	71.5	332	73.8	0.846	0.358	73.8(69.5–77.8)
**Do you have a fear that you could carry the infection from your dental practice back to your family**?	250	94.7	167	89.8	417	92.7	3.874	0.049*	92.7(89.9–94.9)
**Are you afraid of getting quarantined if you get infected?**	204	77.3	132	71.0	336	74.7	2.293	0.130	74.7(70.4–78.6)
**Are you anxious about the cost of treatment if you get infected?**	158	59.8	105	56.5	263	58.4	.518	0.472	58.4(53.7–63)
**Do you feel afraid when you hear that people are dying because of COVID-19?**	227	86.0	148	79.6	375	83.3	3.233	0.072	83.3(79.7–86.7)
**Do you think a surgical mask is enough to prevent cross-infection of COVID-19?**	61	23.1	57	30.6	118	26.2	3.206	0.073	26.2(22.2–30.5)
**Do you think N-95 masks should be routinely worn in dental practice due to the current pandemic?**	219	83.0	152	81.7	371	82.4	0.115	0.735	82.4(78.6–85.8)

*CI* Confidence interval

χ^2^ = chi square test

^*^ Significant

Regarding students^,^ practice, 81.8% followed the universal infection control precautions, and 5th academic year students showed a significantly higher percentage (84.9%) as compared to other grades (*p*=0.030, 95% CI =81.8(77.9-85.2). Approximately two-thirds of students reported the use of high-volume suction in their practice for every patient with aerosol-generating procedures (AGP) (64.4%). This percentage was significantly higher in 4^th^-year academic students (72.4%) followed by 5^th^ year (60.3%) and 3^rd^ year (52.2%), *p*=0.002, 95% CI =64.4(59.8-68.9) (Table 2).

**Table 2:** Practice of the studied students according to their academic year (*n* = 450)

**Questions**	**Academic year** **(Frequency of Yes answers)**								**χ**^**2**^	***P* value**	**95% CI of total** **yes percentage**
	**3**^**rd**^ ***n* = 90**		**4**^**th**^ ***n* = 214**		**5**^**th**^ ***n* = 146**		**Total** ***n* = 450**				
	**n**	**%**	**n**	**%**	**n**	**%**	**n**	**%**			
**Do you always update your knowledge with the current CDC or WHO Guidelines for cross-infection control regarding COVID-19?**	68	75.6	158	73.8	110	75.3	336	74.7	0.152	0.927	74.7(70.4–78.6)
**Do you usually ask every patient’s travel history before performing dental treatment?**	59	65.6	141	65.9	103	70.5	303	67.3	1.019	0.601	67.3(62.8–71.7)
**Do you usually take every patient’s body temperature before performing dental treatment?**	36	40.0	82	38.3	58	39.7	176	39.1	0.110	0.947	39.1(34.6–43.8)
**Are you deferring dental treatment of patients showing suspicious symptoms?**	63	70.0	165	77.1	113	77.4	341	75.8	2.05	0.359	75.8(71.5–79.7)
**Do you usually wear an N-95 mask during patient contact?**	32	35.6	74	34.6	59	40.4	165	36.7	1.33	0.514	36.7(32.2–41.3)
**Do you routinely follow universal/standard precautions of infection control for every patient?**	65	72.2	179	83.6	124	84.9	368	81.8	6.99	0.030*	81.8(77.9–85.2)
**Do you use rubber dam isolation for every patient?**	39	43.3	85	39.7	74	50.7	198	44.0	4.25	0.119	44.0(39.4–48.7)
**Do you use high-volume suction in your practice for every patient with AGP?**	47	52.2	155	72.4	88	60.3	290	64.4	12.9	0.002*	64.4(59.8–68.9)
**Do you ask every patient to rinse his/her mouth with anti-bacterial mouthwash before treatment?**	50	55.6	114	53.3	79	54.1	243	54.0	0.134	0.935	54.0(49.3–58.7)
**Do you wash hands with soap and water/use sanitizer before and after treatment of every patient?**	73	81.1	208	97.2	142	97.3	423	94.0	33.1	0.000*	94.0(91.4–96)
**Are you aware of which authority to contact if you come across a patient with a suspected COVID-19 infection?**	55	61.1	167	78.0	108	74.0	330	73.3	9.32	0.009*	73.3(69.0–77.4)
**Do you use of FFP2/FFP3 facial filter?**	55	61.1	109	50.9	64	43.8	228	50.7	6.66	0.036*	50.7(45.9–55.4)
**Do you use disposable gowns?**	65	72.2	192	89.7	114	78.1	371	82.4	16.2	0.000*	82.4(78.6–85.8)
**Do you use safety glasses or a visor?**	54	60.0	152	71.0	106	72.6	312	69.3	4.71	0.095	69.3(64.8–73.6)
**Do you use a rotating instrument with an anti-retraction valve (handpieces)?**	42	46.7	131	61.2	73	50.0	246	54.7	7.31	0.026*	54.7(49.9–59.3)

*CI* Confidence interval

χ^2^ = chi square test

^*^Significant

Most students** (**94.0%) washed their hands with soap and water or used sanitizer before and after treating each patient with statistically significant differences between different years (*p*=0.000, 95% CI =94.0(91.4-96). Most students (73.3%) maintained awareness about the concerned authority that needed to be approached if they encountered a patient with suspected COVID-19 symptoms. This percentage was significantly higher for 4^th^ year students (78%), *p*=0.009, 95% CI =73.3(69.0-77.4) (Table 2).

More than half of the students (50.7%) used Filtering Face Piece 2/3 (FFP2/FFP3) with a significantly higher percentage for 3^rd^-year students (61.1%), *p* = 0.036, 95% CI = 50.7(45.9–55.4). Most students (82.4%) used disposable gowns and 4^th^-year students showed a higher percentage (89.7%), *p* = 0.000, 95% CI = 82.4(78.6–85.8). More than half of students (54.7%) used rotating instruments with anti-retraction valves (handpiece) with a significantly higher percentage for 4^th^-year students (61.2%), *P* = 0.026, 95% CI = 54.7(49.9–59.3) (Table 2).

**Fig. 1:**
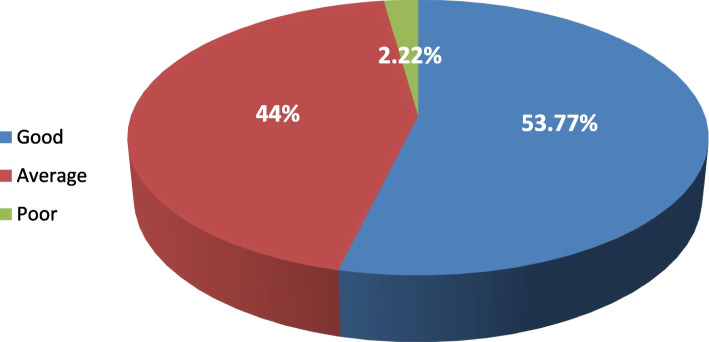
Total practice score of the studied students

**Fig. 2:**
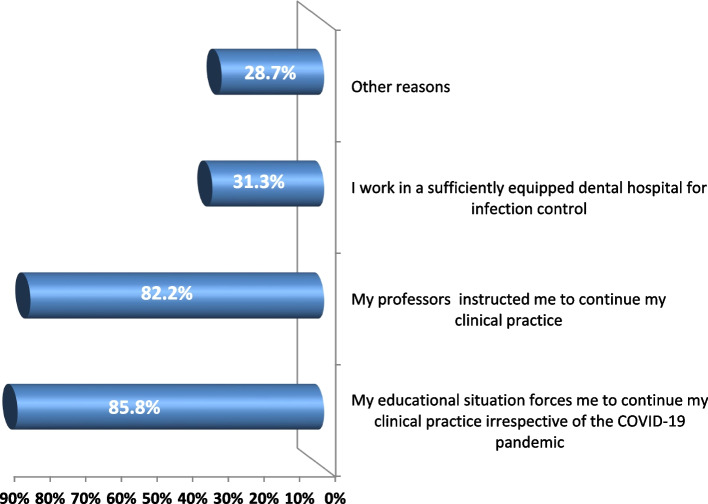
The reasons why students decided to continue their clinical work during the COVID-19 pandemic

More than half of the students (53.7%) had good practice scores, followed by 44% with average practice and 2.2% with poor practice (Fig. 1). The reasons why students decided to continue their clinical work during the COVID-19 pandemic varied where 85.8% reported that their educational situation forced them to continue irrespective of the pandemic. Also, 82.2% of students were instructed by their professors to continue clinical practice. Only one-third of students (31.3%) reported working in a sufficiently equipped hospital for infection control (Fig. 2).

A good practice score was insignificantly associated with the female gender, the urban community and not belonging to the risk group. Meanwhile, there were statistically significant differences between groups regarding age as determined by one–way ANOVA (F = 3.152, *P* = 0.044). Also, the practice score was significantly associated with the academic year, wherein 50% of the 3rd year students had a poor practice score, while 49.6% of the 4th year students demonstrated good practice scores, *p*-value of 0.044 (Table 3).

**Table 3:** Relationship between sociodemographic data and total practice score

**Variable**	**Total practice score**								**Test of significance**	***P*-value**
	**Good (*n* = 242)**		**Average (*n* = 198)**		**Poor (*n* = 10)**		**Total (*n* = 450)**			
	**n**	**%**	**n**	**%**	**n**	**%**	**n**	**%**		
**Age (Years)**									**ANOVA**	
**Mean** ± **SD**	21.79 ± 0.938		21.62 ± 0.969		21.2 ± 1.31		21.70 ± 0.965		F = 3.15	0.044*
**Range**	20–24		20–24		20–24		20–24			
**Gender**										
Female	147	60.7	110	55.6	7	70.0	264	58.7	χ^2^ = 1.751	0.417
Male	95	39.3	88	44.4	3	30.0	186	41.3		
**Residence**										
Urban	142	58.7	110	55.6	5	50.0	257	57.1	χ^2^ = 0.645	0.725
Rural	100	41.3	88	44.4	5	50.0	193	42.9		
**Academic year**										
3^rd^	39	16.1	46	23.2	5	50.0	90	20.0	χ^2^ = 9.771	0.044*
4^th^	120	49.6	92	46.5	2	20.0	214	47.6		
5^th^	83	34.3	60	30.3	3	30.0	146	32.4		
**Belonging to the risk group due to comorbidity**										
Yes	58	24.0	54	27.3	3	30.0	115	25.6	χ^2^ = 0.732	0.694
No	184	76.0	144	72.7	7	70.0	335	74.4		

χ^2^ = chi square test

^*^ Significant

The following factors were identified as significant predictors of a good practice score, based on the results of the logistic regression analysis (Table 4). Students who always update their knowledge with the current guidelines for cross-infection regarding COVID-19 (Exp B = 12.058, *p* = 0.001, 95% CI = 53.20, 2.733). Students who usually ask every patient’s travel history before performing treatment (Exp B 5.216, *p* = 0.021, 95% CI = 20.688, 1.054), deferring dental treatment of patients showing suspicious symptoms (Exp B = 4.669, *p* = 0.042, 95% CI = 21.149, 1.286), following routine universal/ standard precautions of infection for every patient (Exp B = 7.242, *p* = 0.016, 95% CI = 36.469, 1.438), using high-volume suction for every patient (Exp B = 5.054, *p* = 0.025, 95% CI = 20.826, 1.226) and using safety glasses or visor (Exp B = 4.896, *p* = 0.036, 95% CI = 21.673, 1.106).

**Table 4:** logistic regression for factors associated with good practice score level a

**Good practice**	**B**	**Wald**	**Sig**	**Exp(B)**	**95% Confidence Interval for Exp(B)**	
					Lower	Lower
**Intercept**	-79.424-	43.28	**0.000**			
**Always update my knowledge with the current guidelines for cross-infection regarding COVID-19 (yes)**	2.490	10.80	0.001*	12.058	2.733	53.202
**Usually ask every patient’s travel history before performing treatment(yes)**	1.652	5.348	0.021*	5.216	1.286	21.149
**Deferring dental treatment of patients showing suspicious symptoms(yes)**	1.541	4.116	0.042*	4.669	1.054	20.688
**Follow routine universal precautions for infection for every patient(yes)**	1.980	5.762	0.016*	7.242	1.438	36.469
**Using high-volume suction for every patient(yes)**	1.620	5.028	0.025*	5.054	1.226	20.826
**Using safety glasses or visor(yes)**	1.588	4.379	0.036*	4.896	1.106	21.673

^a^Reference category is poor practice

^*^Significant

## Discussion

In dental universities, a considerable portion of patient care is managed by students as part of their clinical course; this puts them at risk of infection.

In the current study, the participants' ages ranged from 20 to 24 years, and more than half of the students were females. Also, an Iranian study in 2023 reported that 62.4% of students were females [27]. More than half of the students in the current study were from the urban community, which agrees with the results of a study in Palestine in 2021. However, the proportion of students in the risk group for coronavirus infection in the study mentioned above was considerably lower (3.3%) than that in the current study (25%) [28].

In the present study, most dental students feared getting infected by their patients or co-workers and feared treating patients with suspicious symptoms (Table 1). Another Egyptian study in 2020 reported that 92.6% of studied professionals were anxious about becoming infected with COVID-19 [8]. A study in Texas in 2022 reported that 77% were concerned about the impact of COVID-19 on their general health, safety, and well-being [29]. The knowledge that millions of people worldwide were affected by this fatal virus and either isolated/ quarantined or died could result in considerable psychological stress and fear [30].

The current study showed that most of the students were fearful of providing treatment to patients with suspicious symptoms (Table 1), similarly Egyptian study 2020 reported that 90.7% feared treating patients with suspicious symptoms [8]. This was justified, owing to the sharp increase in COVID-19 cases in almost every country across the world [31]. The use of telehealth activities will aid in alleviating this risk by minimizing the number of face-to-face interactions. The WHO cited telemedicine as one of the essential services for "strengthening the Health Systems Response to COVID-19" policy [32].

We found that the vast majority of the students in this study were worried about carrying the infection back to their families (Table 1).In accordance with a Malaysian study in 2021, 89.3% of respondents were concerned about their family's health [33]. Also Consistent to a study in China, college students were more worried about their elder family members [34]. The prolonged incubation period of the coronavirus before the appearance of the symptoms, along with the fact that it can be present on various surfaces for a few hours to up to a few days, make it challenging to limit the spread of this disease [35].

Our study also revealed that risk perception was significantly higher among females as compared to males concerning the above-mentioned risks similar to the Malaysian study 2020 among medical and dental students [36]. Higher risk perceptions among females can be described by their unfeasible thoughts, gender socialization, and high sensitivity toward health risks [34].

About three-quarters of the students feared being quarantined (Table 1). In agreement with the Egyptian study 2020, 69.9% is the same [8]. More than half were anxious about the cost of treatment (if infected). This can be explained by inadequacies in the health insurance system that have been reported worldwide, including in Egypt, which could result in a significant financial burden on the patient. Critical COVID-19 patients often require expensive treatments, such as mechanical ventilation and extracorporeal membrane oxygenation, directly increasing healthcare costs [37].

We found that 83.3% expressed discomfort and fear after hearing about the deaths of people due to COVID-19 while the Egyptian study in 2020 reported a lower percentage 71.3% [8].

We reported about two-thirds wanted to continue with the clinical procedures once the number of COVID-19 cases had declined. Similarly, an Egyptian study in 2020 reported that 69.9% wanted the same [8].

We concluded that 73.8% felt nervous while talking to the patients without maintaining social distancing. Similarly, an Egyptian study in 2020 reported that 72.2% was the same [8].

Dental students' perceived risks increased due to the novel nature of the virus, contact with untested patients, the high risk of inhaling saliva and respiratory droplets in the small dental unit, and patients' lack of awareness of the condition and safety precautions [21, 38].

Overwhelming feelings of fear, confusion, and anxiety among dental students in the current study will be implicated in a sudden decrease in the number of performed dental procedures [4].

Concerning students' practice, generally, students performed good preventive behaviors. It may be due to better-perceived susceptibility and an increase in dental students’ perceived risks [39]. There was a significant association between the academic year of the students and their practice regarding universal/standard precautions, high-volume suction, washing hands with soap and water, authority to contact, FFP2/FFP3, disposable gowns, and hand pieces (Table 2). In accordance with the Saudi Arabian study 2021 [40].

The majority of the students practiced washing their hands with soap and water and using a sanitizer before and after treating each patient, using a disposable gown, and following the universal and standard infection control protocol (Table 2) in accordance with the studies by Mahdee et al. and Tarakji et al. [41, 42].

In the current study, 74.7% of the students responded positively concerning updating their knowledge about the current CDC or WHO guidelines for cross-infection, in accordance with Ahmed et al. 2020 who reported a higher percentage of 90% [23].

In the present series, about seventy percent were aware of the concerned authority that must be approached if they came across a patient with suspected COVID-19 infection (Table 2). Similar findings were reported in other studies [23, 43].

More than 70% of the students responded positively concerning deferring the dental treatment of patients with suspicious symptoms (Table 2). In agreement with Ahmed et al. 2020 [23]. In the present study, about two-thirds enquired about the patient’s travel history before performing any procedure (Table 2). Similarly, more than 60% confirmed that in the Saudi Arabian study 2021 [42]. Also, Ahmed et al. 2020 reported a higher percentage (82%) [23].

In the current study, about half of the students instructed every patient to rinse their mouth with an anti-bacterial mouthwash before treatment, use FFP2/FFP3, and rotate instruments with anti-retraction valves (Table 2). In contrast, The number of students who used an FFP2/FFP3 facial filter was higher (94%) in a Mexican study in 2021 [44]. This difference may be due to the limited supply of the filters in the Egyptian government universities.

In the current study, 39% measured the body temperature of each patient before performing a dental procedure and 44% used a rubber dam (Table 3). A Saudi Arabian study in 2021 reported a higher rate (88.7%) of dentists who recorded the patient's body temperature and a lower rate of using a rubber dam [42]. However, it is worth noting that the Saudi Arabian study comprised dentists rather than students, who needed to be more experienced.

In the current study, the lowest practice score was related to wearing an N-95 mask during patient contact (Table 2), which may be due to the limited supply of the mask owing to its high cost compared to other types of masks. Meanwhile, Ahmed et al. 2020 reported a much lower percentage (9%) [23].

Implication for the partial commitment of our students to using mouthwash, FFP2/FFP3, rotating tools, not assessing patients' body temperatures, not using a rubber dam and N-95 mask, they are exposed to high infection rates. Dentists should adhere to strong personal protection precautions, personal safety gear, hand washing, thorough patient assessment, rubber dam isolation, anti-retraction (handpiece), mouth rinse before dental treatments and clinic disinfection [16].

The present study showed that the practices of most students in the current study ranged from good to average and were significantly associated with the academic year (Fig. 1). Similarly, a multinational study in 2020 recorded the same results [45]. In contrast to Malaysian study in 2021 reported no significant difference in the mean preventive behavior scores when comparisons were made between students across years and phases of study [33].

There are challenges facing Egyptian students that may be responsible for this diversity in their practice especially after the majority of Egyptian dental programs adopted the hybrid learning model, which limits in-person learning to laboratory and clinical courses following the regulations of the Egyptian Supreme Council of Universities. This tactic was used to reduce physical contact [46].

Resources and thorough critical preparation were major challenges needed to teach theoretical lecturers. For instance, having a reliable internet connection is necessary to offer online educational services, which could be a problem for some developing nations. In light of the large numbers of Egyptian students in the college, social distance in classes (safe environment), scheduling adjustments, and newly mandated public health regulations were other challenges [46].

Lack of personal protective equipment, comfort or discomfort, and compatibility with specialized dental equipment were further issues [47].

In the present study, about one-third of the students wanted to continue their clinical course during the COVID-19 pandemic as they believed they worked in a sufficiently equipped dental hospital for infection control (Fig. 2). A higher percentage (95%) were satisfied with the resources their dental practice provided during the pandemic in Texas in 2022 [29].

In many parts of the country, the pandemic made it difficult for students in the class of 2020 to complete their graduation requirements during March, April, and May 2020. In dental school clinics and extramural rotations, students directly caring for patients raised serious concerns. Due to the COVID-19 epidemic, the Commission on Dental Accreditation permitted changes to clinical standards and procedures. A virtual clinical curriculum was immediately adopted by several dental universities. The emphasis was placed on case presentations and group discussions regarding case management. Numerous qualifications and skills were fulfilled virtually, and several clinical requirements were satisfied after local state laws had already made elective dental care legal. Exams for dental licensing were also modified as a result of the pandemic from live-patient to mannequin-based. Virtual modalities were also adopted for graduation exercises, externships, and interviews for dental residency programs [48].

Students who always update their knowledge with the current guidelines for cross-infection regarding COVID-19, students who usually ask every patient’s travel history before performing treatment, deferring dental treatment of patients showing suspicious symptoms, following routine universal/ standard precautions of infection for every patient, using high-volume suction for every patient with AGP and using safety glasses or visor were identified as significant predictors of good practice (Table 4). Similar to the findings of a Turkish study in 2020 [49].

A Lebanese study in 2020 revealed that a high level of knowledge, trained and specialist dentists had better preventive practice regarding COVID-19. Moreover, fear of treating COVID-19 patients is associated with poor practice [50].

## Limitations

This study portrayed some important psychological impacts of COVID-19 on dental students and some practice responses to these issues. However, using a convenience sample was a limitation and a source of selection bias but we were obliged to due to the hybrid training system we tried to approach participants as much as we could to complete the required sample size. In addition, self-reported data is another limitation, but an important objective of this study was to assess the risk perception of participants which necessitated their self-reporting.

## Conclusion

The SARS-CoV-2 pandemic has caused a paradigm shift in how dental academic institutions are operating. A significant proportion of dental students were worried about contracting an infection while doing dental care. Some of them frequently accepted the increased risk of infection as a requirement for graduation and made changes to their dental practices in accordance with the advised standards. Others want to temporarily halt their dental practices till the incidence of COVID-19 patients starts to decline. The majority of dental students knew the most recent CDC or WHO recommendations for preventing cross-infection.

To conclude, Dental students at Tanta University exhibited anxiety and fear while caring for their patients owing to the COVID-19 pandemic. Moreover, considerable changes in their dental behavior/practices were observed following the standard guidelines.

## Recommendations

Based on these findings, providing psychological support for dental students and enabling them to deal with such pandemics without fear is recommended.

Regular online and offline infection control sessions to update students' knowledge regarding COVID-19 infection are highly recommended.

It is strongly advised that all dental schools have student counselors who are accessible for in-person and online counseling sessions. Peer support is a great approach to spot problems with stress and anxiety in pupils and start solving them.

To ensure a safe dental environment, several online platforms should be used. Virtual learning should replace clinical learning. Haptic technologies, virtual/augmented reality simulation devices, and manikins can all be highly beneficial for learning new skills.

According to the new infection control guidelines, educational clinical dental settings must be changed as soon as feasible to enable high-quality instruction for dental students and the continuation of community dental services in a secure setting.

Future research can explore the different psychological effects of COVID-19 using specific tools and the effect of psychological support interventions for dental students. In addition, quantifying the risk of different dental procedures to prioritize them according to the probability of spreading infection is needed.

The pedagogical impacts of the structural change in dental educational methods brought on by the COVID-19 crisis will need to be assessed in the future. Dental schools must draw lessons from this experience and keep in mind the necessity of sharing information and adhering to strict professional obligations. It's important to provide answers to several issues about dental clinical education that could aid with knowing what might be done in the future, such as what fundamental infection control standards will exist following the COVID-19 immunization. The positioning, spacing, and central air ventilation of dental units may require future revisions to the architecture and infrastructure of teaching clinics, including patient waiting spaces. Plans for institutional assistance for students, teachers, and staff are essential to assist them in coping with the negative effects of the current crisis.

## Data Availability

The datasets generated and analyzed during the current study are not publicly available. However, the datasets are available from the corresponding author on a reasonable request.

## References

[1] Hamid H, Khurshid Z, Adanir N, Zafar MS, Zohaib S (2020). COVID-19 pandemic and role of human saliva as a testing biofluid in point-of-care technology. Eur J Dent.

[2] Khachfe HH, Chahrour M, Sammouri J, Salhab H, Makki BE, Fares M. An Epidemiological Study on COVID-19: A rapidly spreading disease. Cureus. 2020;12(3):e7313–21. 10.7759/cureus.7313. 10.7759/cureus.7313 PMC7164711 32313754

[3] Haileamlak A (2021). The impact of COVID-19 on health and health systems. Ethiop J Health Sci.

[4] Tysiąc-Miśta M, Dziedzic A. The attitudes and professional approaches of dental Practitioners during the COVID-19 Outbreak in Poland: a cross-sectional survey. Int J Environ Res Public Health. 2020;17(13):4703–18.10.3390/ijerph17134703. 10.3390/ijerph17134703 PMC7370196 32629915

[5] Cao W, Fang Z, Hou G, Han M, Xu X, Dong J (2020). The psychological impact of the COVID-19 epidemic on college students in China. Psychiatry Res.

[6] Kang L, Ma S, Chen M, Yang J, Wang Y, Li R (2020). Impact on mental health and perceptions of psychological care among medical and nursing staff in Wuhan during the 2019 novel coronavirus disease outbreak: a cross-sectional study. Brain Behav Immun.

[7] Almalki AH, Alzahrani MS, Alshehri FS, Alharbi A, Alkhudaydi SF, Alshahrani RS (2021). The psychological impact of COVID-19 on healthcare workers in Saudi Arabia: a year later into the pandemic. Front Psych.

[8] Aly MM, Elchaghaby MA (2020). Impact of novel coronavirus disease (COVID-19) on Egyptian dentists’ fear and dental practice (a cross-sectional survey). BDJ Open.

[9] World Health Organization. Egypt: WHO coronavirus disease (COVID-19) dashboard with vaccination data. Available from: https://covid19.who.int/region/emro/country/eg. Acccessed 7 Oct 2022.

[10] Epstein JB, Chow K, Mathias R (2021). Dental procedure aerosols and COVID-19. Lancet Infect Dis.

[11] Sabino-Silva R, Jardim ACG, Siqueira WL (2020). Coronavirus COVID-19 impacts to dentistry and potential salivary diagnosis. Clin Oral Invest.

[12] Amato A, Caggiano M, Amato M, Moccia G, Capunzo M, De Caro F (2020). Infection control in dental practice during the COVID-19 pandemic. Int J Environ Res Public Health.

[13] Guo H, Zhou Y, Liu X, Tan J. The impact of the COVID-19 epidemic on the utilization of emergency dental services. J Dent Sci. 2020;15(4):564–7. 10.1016/j.jds.2020.02.002. 10.1016/j.jds.2020.02.002 PMC7156222 32296495

[14] Occupational Safety and Health Administration. COVID-19 control and prevention: dentistry workers and employers. U.S. DEPARTMENT OF LABOR, Occupational Safety and Health Administration 200 Constitution Ave NW, Washington, DC 20210. https://www.osha.gov/coronavirus/control-prevention/dentistry.

[15] American Dental Association. https://www.dentistry33.com/clinical-cases/oral-hygiene-prevention/222/american-dental-association-ada-recommendations-on-the-treatment-of-urgency-and-emergency-cases-in-dental-practices.html. Accessed 7 Oct 2022.

[16] World Health Organization: Clinical management of severe acute respiratory infection when novel coronavirus (nCoV) infection is suspected Interim guidance, 12 January 2020. https://www.who.int/publications/i/item/10665-332299.

[17] Cianetti S, Pagano S, Nardone M, Lombardo G (2020). Model for taking care of patients with early childhood caries during the SARS-Cov-2 pandemic. Int J Environ Res Public Health.

[18] Ataş O, Talo Yildirim T (2020). Evaluation of knowledge, attitudes, and clinical education of dental students about COVID-19 pandemic. PeerJ..

[19] Turkistani KA, Turkistani KA (2020). Dental risks and precautions during COVID-19 pandemic: a systematic review. J Int Soc Prev Commun Dent.

[20] Hung M, Lipsky MS, Phuatrakoon TN, Nguyen M, Licari FW, Unni EJ (2022). Teledentistry Implementation During the COVID-19 Pandemic: Scoping Review. Interact J Med Res..

[21] Alawia R, Riad A, Kateeb E (2022). Risk perception and readiness of dental students to treat patients amid COVID-19: Implication for dental education. Oral Dis.

[22] Nair A, Singla N, Singla R, De A. Risk Perception and Preparedness of Undergraduate Dental Students to Treat Patients in View of COVID-19 Pandemic: A questionnaire survey. ScientificWorldJournal. 2022;2022:Article ID 4489773. 10.1155/2022/4489773. 10.1155/2022/4489773 PMC9797310 36590926

[23] Ahmed MA, Jouhar R, Ahmed N, Adnan S, Aftab M, Zafar MS (2020). Fear and practice modifications among dentists to combat novel coronavirus disease (COVID-19) outbreak. Int J Environ Res Public Health.

[24] Prevention II. Control Guidance for Dental Settings During the Coronavirus Disease 2019 (COVID-19) Pandemic (2020). Guidance for Dental Settings Centers for Disease Control and Prevention.

[25] de Odontologia CF. Manual de boas práticas em biossegurança para ambientes odontológicos. CFO: Brasília. 2020.

[26] Hashemzaei M, Afshari M, Koohkan Z (2021). Knowledge, attitude, and practice of pharmacy and medical students regarding self-medication, a study in Zabol University of Medical Sciences; Sistan and Baluchestan province in south-east of Iran. BMC Med Educ..

[27] Shahroudi AS, Hashemikamangar S-S, Aljawad ZAA, Behniafar B (2023). Dental students’ knowledge about protective guidelines for clinical practice during the COVID-19 pandemic. J Oral Biol Craniofac Res.

[28] Salameh B, Basha S, Basha W, Abdallah J (2021). Knowledge, Perceptions, and Prevention Practices among Palestinian University Students during the COVID-19 Pandemic: A Questionnaire-Based Survey. INQUIRY.

[29] Bsoul EA, Challa SN, Loomer PM (2022). Multifaceted impact of COVID-19 on dental practice: American dental care professionals prepared and ready during unprecedented challenges. J Am Dent Assoc.

[30] Oyat FWD, Oloya JN, Atim P, Ikoona EN, Aloyo J, Kitara DL. The psychological impact, risk factors and coping strategies to COVID-19 pandemic on healthcare workers in the sub-Saharan Africa: a narrative review of existing literature. BMC Psychol. 2022;10(1):284. 10.1186/s40359-022-00998-z. 10.1186/s40359-022-00998-z PMC9714392 36457038

[31] Wong J, Lee AHC, Zhang C. Effect of COVID-19 on dental education and endodontic practice in Hong Kong. Front Dent Med. 2020;1:569225–5. 10.3389/fdmed.2020.569225.

[32] Ghai S (2020). Teledentistry during COVID-19 pandemic. Diabetes Metab Syndr.

[33] Ismail A, Ismail NH, Abu Kassim NYM, Lestari W, Ismail AF, Sukotjo C (2021). Knowledge, perceived risk, and preventive behaviors amidst Covid-19 pandemic among dental students in Malaysia. Dent J.

[34] Ding Y, Du X, Li Q, Zhang M, Zhang Q, Tan X (2020). Risk perception of coronavirus disease 2019 (COVID-19) and its related factors among college students in China during quarantine. PLoS One.

[35] Van Doremalen N, Bushmaker T, Morris DH, Holbrook MG, Gamble A, Williamson BN (2020). Aerosol and surface stability of SARS-CoV-2 as compared with SARS-CoV-1. N Engl J Med.

[36] Wee EG, Giri MS, Sundram TK, Venudran CV (2020). COVID-19: Knowledge, attitude and preventive behaviors of medical and dental students. Int J Biomed Clin Sci.

[37] Jin H, Wang H, Li X, Zheng W, Ye S, Zhang S (2021). Economic burden of COVID-19, China, January–March, 2020: a cost-of-illness study. Bull World Health Organ.

[38] Jum’ah AA, Elsalem L, Loch C, Schwass D, Brunton PA (2021). Perception of health and educational risks amongst dental students and educators in the era of COVID-19. Eur J Dental Educ.

[39] Barakat AM, Kasemy ZA (2020). Preventive health behaviours during coronavirus disease 2019 pandemic based on health belief model among Egyptians. Middle East Curr Psychiatry.

[40] Bin Mubayrik A, Al Dosary S, Alwasil W, AlShanqeeti B, Alkathiri M, Alahmari R, et al. Knowledge and practice of COVID-19 infection control among dental students and interns: a cross-sectional survey. Adva Medi EducPract. 2021:1419–27. 10.2147/AMEP.S345713 PMC8664341 34908890

[41] Mahdee AF, Gul SS, Abdulkareem AA, Qasim SSB. Anxiety, practice modification, and economic impact among iraqi dentists during the COVID-19 outbreak. Front Med. 2020;7. 10.3389/fmed.2020.595028 PMC7793761 33425944

[42] Tarakji B, Nassani MZ, Alali FMB, Alsalhani A, Alqhtani NR, Bin Nabhan A (2021). COVID-19—awareness and practice of dentists in Saudi Arabia. Int J Environ Res Public Health..

[43] Aly MM, Elchaghaby MA (2020). Impact of novel coronavirus disease (COVID-19) on Egyptian dentists’ fear and dental practice (a cross-sectional survey). BDJ Open.

[44] Gómez-Clavel JF, Morales-Pérez MA, Argumedo G, Trejo-Iriarte CG, García-Muñoz A, editors. Concerns, Knowledge, and Practices of Dentists in Mexico Regarding Infection Control during the Coronavirus Disease Pandemic: A Cross-Sectional Study. Healthcare; 2021: Multidisciplinary Digital Publishing Institute. 10.3390/healthcare9060731 PMC8231957 34198601

[45] Kamate SK, Sharma S, Thakar S, Srivastava D, Sengupta K, Hadi AJ (2020). Assessing Knowledge, Attitudes and Practices of dental practitioners regarding the COVID-19 pandemic: a multinational study. Dent Med Probl.

[46] Hassan MG, Amer H (2021). Dental Education in the Time of COVID-19 Pandemic: Challenges and Recommendations. Front Med..

[47] Vogt LC, Reske KA, Park D, Habrock Bach T, Stewart HB, Arter OG (2023). Personal protective equipment use among dental healthcare personnel during the coronavirus disease 2019 (COVID-19) pandemic and the impact of an educational video in clinical practice. Infect Control Hosp Epidemiol..

[48] Panchal N, Wolff M. The profound impact of COVID-19 on the dental profession. Compendium. 2021;42(6). 34077665

[49] Özdede M, Sahin S (2020). Views and anxiety levels of Turkish dental students during the COVID-19 pandemic. J Stomatol.

[50] Nasser Z, Fares Y, Daoud R, Abou-Abbas L (2020). Assessment of knowledge and practice of dentists towards Coronavirus Disease (COVID-19): a cross-sectional survey from Lebanon. BMC Oral Health.

